# Equal cardiac arrest care — a qualitative study of healthcare professionals’ experiences

**DOI:** 10.1186/s12910-026-01428-0

**Published:** 2026-03-05

**Authors:** Liselott Årestedt, Johan Israelsson, Jens Agerström, Kristofer Årestedt, Anders Bremer

**Affiliations:** 1 Department of Health and Caring Sciences, Faculty of Health and Life Sciences, Linnaeus University, Kalmar, Växjö, Sweden; 2 Department of Internal Medicine, Division of Cardiology, Kalmar County Hospital, Kalmar, Sweden; 3 Department of Psychology, Faculty of Health and Life Sciences, Linnaeus University, Kalmar, Växjö, Sweden; 4 Department of Research, Region Kalmar County, Kalmar, Sweden

**Keywords:** Cardiopulmonary resuscitation, Equal care, Healthcare professionals, Heart arrest, Qualitative research, Thematic analysis

## Abstract

**Background:**

Discrimination and inequities in healthcare have gained increased attention worldwide. Although many healthcare systems strive to provide equal care, evidence indicates that treatment can be influenced by patient characteristics such as gender, age, race/ethnicity, and socioeconomic status. In particular, research exploring healthcare professionals’ perceptions on equality in acute, life-threatening conditions remains limited. This study aimed to describe Swedish healthcare professionals’ perceptions of unequal and equal care when treating patients with cardiac arrest.

**Methods:**

The study adopted a qualitative inductive design, using semi-structured interviews with 12 physicians and nurses who work in acute care clinics in Sweden. Data were analysed using thematic analysis.

**Results:**

The overarching theme, “Equal care under pressure—guided by principles, shaped by context”, captured how professionals perceive the provision of cardiopulonary resuscitation (CPR) during sudden cardiac arrest as fundamentally equal. CPR was described as an automatic, protocol-driven intervention offered to all patients, reinforcing the principle of equality and without any clear discrimination based on ethnicity, gender, or socioeconomic background. However, equality in practice was nuanced. Decision-making about CPR introduce ethical complexity, particularly for older patients from whom prognostic uncertainty and emotional factors could influence care. Team competence was critical for maintaining equity under pressure, while contextual factors such as location, cultural norms, and safety concerns could delay or complicate treatment. Finally, professionals emphasize the need for structured reflection, recognizing its role in learning and ethical decision-making. Together, these findings illustrate that while CPR was guided by principles of equal care, its delivery was shaped by clinical judgment, team dynamics, and situational realities.

**Conclusions:**

Healthcare professionals perceive cardiac arrest care as predominantly equal. However, subtle vulnerabilities challenge this perception. Older age emerged as the most influential factor in CPR decisions, raising concerns about potential ageism and the need for clearer Do Not Attempt Resuscitation (DNAR) criteria and patient involvement. Promoting equality requires team competence, structured reflection, and proactive communication about resuscitation preferences. Further research should examine age, multimorbidity, psychiatric illness, DNAR practices, and gender differences to strengthen equity in cardiac arrest care.

**Supplementary Information:**

The online version contains supplementary material available at 10.1186/s12910-026-01428-0.

## Background

Challenges related to discrimination and equal healthcare have received increasing attention in recent years [1–3]. In the healthcare settings, discrimination can manifest through the opinions, beliefs, behaviours, and attitudes of healthcare professionals (HCPs), leading to differences in how patients from diverse social groups are received and treated [4–7]. Discrimination occurs when inequality between individuals arises based on illness, disability, religion, sexual orientation, or other dimensions of diversity [8]. Such disparities threaten the fundamental principles of justice and equality in healthcare.

Cardiac arrest care represents a relatively new area of research within the broader discourse on equitable healthcare [9]. Existing studies reveal disparities in treatment and outcomes across several social dimensions. For example, gender differences have been documented: men are more likely to receive resuscitation attempts during in-hospital cardiac arrest, yet women appear to have higher survival rates [10–12]. A systematic review of out-of-hospital cardiac arrests found that women had a survival advantage despite receiving fewer advanced interventions such as coronary angiography, percutaneous coronary angiography, and targeted temperature management therapy [13].

Age-related discrimination—or ageism—also intersects with other marginalized identities [14]. An American national poll reported that 93.4% of adults aged 50–80 experienced “everyday ageism” [15]. In cardiac arrest care, older patients (> 65 years) receive less basic life support initiation, shorter advanced CPR duration, fewer automated chest compressions, less ventilation, and less epinephrine injections compared to younger patients [16].

Ethnicity and socioeconomic status (SES) further influence cardiac arrest care. North American studies show racial disparities: Black patients admitted during cardiac arrest have lower survival rates than White patients, even after adjusting for hospital factors [17, 18]. However, findings are not uniform; a Swedish registry study found no significant differences in treatment for Middle Eastern and African patients compared to Nordic patients and even reported higher survival among Middle Eastern patients [19].

SES-related differences have also been observed: patients from lower SES backgrounds are less likely to receive bystander CPR and have lower survival rates [20–23], although some studies report no association [24]. These inconsistencies suggest that discrimination may be context-dependent, varying across cultures and welfare systems. Swedish registry data indicate that in-hospital patients with high SES are more likely to receive timely CPR, survive to discharge with good neurological outcome, and receive prophylactic monitoring [9].

In summary, research has documented disparities in cardiac arrest care related to gender, age, ethnicity, and SES, primarily through quantitative analyses of patient outcomes. An American study aimed to shed light on the multifaceted dimensions of HCPs’ understanding of the causes of racial disparities in healthcare. The results indicated notable variation in the degree of importance that HCPs attributed to these causal factors. Those who believed that HCPs contribute to disparities discussed race and racism more readily, identified the mechanisms by which disparities arise, and contextualized patient-level factors more than those who believed that HCPs contributed less to disparities. Differences in understanding the underlying causal factors suggest a multidimensional approach to engaging HCPs in health equity efforts [25]. Qualitative studies have explored discrimination in healthcare more broadly, highlighting how HCPs’ attitudes and contextual pressure can contribute to inequities [25, 26]. For instance, studies describe a “culture of discrimination” shaped by resource constraints, organizational norms, and interpersonal expectations, sometimes forcing HCPs into ethically challenging decisions [27, 28]. However, no qualitative study has examined how HCPs themselves perceive and rationalize equal or unequal care in the specific context of cardiac arrest, i.e., a high-stakes, time-critical setting where decisions are made under pressure and biases may manifest strongly.

Understanding these perceptions is essential for identifying implicit biases, contextual factors, and cultural norms that influence decision-making in emergency care. By situating this study at the intersection of health equity and acute cardiac care, we aim to generate insights that can inform interventions to promote fair and just treatment for all patients. Therefore, the aim of this study was to describe Swedish HCPs’ perceptions of unequal and equal care when treating patients with cardiac arrest.

## Methods

### Design

The study employed a qualitative inductive design, with interviews analysed with reflexive thematic analysis [29, 30]. The study followed the consolidated criteria for reporting qualitative research (COREQ) checklist [31]. The study conforms with the World Medical Association’s ethical principles and ethical approval was received from the Regional Ethical Review Board of Linköping, Sweden (No. 2017/293 − 31). The participants received verbal and written information about the study, including voluntary participation. Written, informed consent was obtained prior to the interviews.

### Setting and participants

A purposive sample was recruited in a county hospital in the south of Sweden. The hospital employs approximately 2,400 staff and has 270 inpatient beds. It comprises departments such as internal medicine and related specialties (e.g., rheumatology and infectious diseases), psychiatry, surgical specialties, anaesthesiology and intensive care, as well as emergency medicine. The Ambulance Service is also based at the hospital. Located in the region’s largest city, the hospital serves a population of about 246,000 inhabitants, with an average age of 45 years—the second highest in Sweden. The region has a population density of 22 inhabitants per square kilometer. The inclusion criteria were Swedish-speaking nurses and physicians who worked in acute care clinics (ambulance care, intensive/anaesthesia care, and cardiology care) and had experience performing CPR. The heads of department at these clinics were informed about the study and gave their consent. Suitable HCPs were identified by a designated nurse and then contacted by email by the first and the last authors. Two persons refused to participate due to a heavy workload, and there were no other dropouts. In total, eight nurses and four physicians agreed to participate.

### Data collection

The 12 interviews were conducted from November 2018 to March 2022. The data collection period was prolonged due to the COVID-19 pandemic when HCPs in acute care were heavily burdened and not available for interviews. The interviews, each held at a single occasion, were carried out at a time and a place convenient for the participants and were performed by the first and the last authors, both experienced researchers in qualitative methods. The first author (LÅ) is a senior lecturer in caring science and she has a background as a registered nurse. The last author (AB) is a professor in caring science and he has a background as an ambulance nurse. Neither of them has any relationship with the participants other than as researchers. Five interviews were conducted digitally via a communication platform with audio recording, and seven were conducted face-to-face. All but one took place at the participants’ workplace, with one at the university, and all were held in private with no other persons present. An interview guide was developed by the team (Supplementary file 1). Each interview started with an open-ended question, “Can you tell us about your experiences caring for patients with sudden cardiac arrest?” with follow-up questions related to some grounds for discrimination found in previous research: gender, age, ethnicity, and socioeconomic status. Data collection was guided by information power, and the 12 interviews were considered sufficient to address the study aim. The interviews were audio-recorded and no field notes were taken. The interviews lasted from 34 to 75 min and were transcribed verbatim by an authorized transcription service.

### Data analysis

Data were analysed, by LÅ and AB, using reflexive thematic analysis according to Braun and Clarke [27, 28]. The analysis process included six phases to identify patterns and develop an understanding of the topic. The transcribed interview texts were read several times to obtain an overall understanding (phase 1) and to generate initial codes (phase 2). All data management and manual coding were conducted in Microsoft Word. In the third phase, potential codes were collated to develop potential themes. Then the themes were reviewed (phase 4) and defined and named (phase 5). In the final step the overarching theme was developed.

## Results

The results consist of one overarching theme and five sub-themes, based on 12 interviews with HCPs (eight nurses and four physicians). Participants characteristics are presented in Table 1.

**Table 1:** Characteristics of participants

Sex, *n*	
Female	9
Male	3
*Age*,* Mdn (range)*	42 (27–66)
*Profession*,* n*	
Nurse	4
Nurse, ambulance	1
Nurse, anaesthesia	2
Nurse, ambulance and anaesthesia	1
Physician	1
Physician, cardiology	2
Physician, anaesthesiologist	1
*Workplace*,* n*	
Ambulance services	2
Anaesthesia/Intensive care unit	3
Cardiac clinic	7

### Equal care under pressure—guided by principles, shaped by context

This overarching theme reflects HCPs’ perception that cardiac arrest care is fundamentally equal, driven by strict guidelines and an ethical commitment to treat all patients. However, their experiences revealed nuances in decision-making, communication, and reflection that influence how equality is enacted in practice. Five sub-themes illustrate these dimensions: (1) Start CPR – “It doesn’t matter who is lying there”, emphasizing universal initiation of resuscitation; (2) Continue or terminate CPR – “What everyone deserves despite multimorbidity”, highlighting dilemmas related to age and comorbidity; (3) Team decisions and communication – “Talking out loud to each other in the team”, underscoring the role of collective competence and open dialogue; (4) The importance of context – “The place contributes a lot”, showing how setting and resources shape care; and (5) Reflection is needed – “Unfortunately you don’t really have the time”, pointing to the value and challenges of post-event reflection.

### Start CPR – “It doesn’t matter who is lying there”

CPR was perceived as one of the most standardized and egalitarian interventions in emergency care. In the context of sudden cardiac arrest, HCPs viewed CPR as an automatic response guided by protocols, leaving little room for subjective judgment or bias. The act of starting CPR symbolized equal treatment, which means that every patient is approached with the same urgency and commitment, regardless of personal characteristics such as gender, ethnicity, or socioeconomic status.

*“I hope most people think like me*,* that it’s a patient on the floor or in bed*,* and that it’s not a dark-skinned person*,* not a white*,* not a Muslim*,* a Jew*,* a Christian*,* but it’s a patient*,* a human // I do CPR until someone tells me not to do CPR because that’s my job. You can have an opinion about it*,* but it doesn’t matter. But I think I speak for most of my colleagues here when I it’s a human being on the floor and that’s all there is to it.”* (Interview 4).

The only recognized exception was the presence of a documented DNAR order, which introduced complexity around advance care planning. While CPR itself was perceived as equal, professionals acknowledged that discussions about CPR and DNAR decisions often occured too late, particularly for older or multimorbid patients. These conversations were considered as ethically important but emotionally demanding; without them unnecessary interventions and suffering may occur.

*“Because either I´m not allowed [to care] for the woman or maybe it’s her husband or another man who doesn’t allow me*,* as a man*,* to do what I’m supposed to do// But it’s hard*,* it’s really hard sometimes // A lot to handle.”* (Interview 1).

In pre-hospital settings, equality was reinforced by collaborative decision-making and a “start unless certain” principle. However, situational factors such as cultural norms around bodily exposure or safety concerns in high-risk environments might have delayed initiation, revealing that equality in principle could be challenged by practical realities.

### Continue or terminate CPR – “What everyone deserves despite multimorbidity”

Decisions about how long CPR should continue reflected a balance between clinical judgment and ethical considerations. While CPR was initiated for all patients, its duration was shaped by medical assessments of prognosis, physiological indicators, and professional expertise. These decisions were framed as objective and evidence-based, yet they occured within a context where age, frailty, and multimorbidity often influenced perceptions of benefit and futility.

Underlying this process was an ethical dilemma: striving to provide what every patient deserved while avoiding unnecessary suffering or overtreatment. Older age and complex illness could introduce risks of unequal care, either through shorter CPR duration or through DNAR decisions made without adequate patient involvement. However, HCPs shared various examples of how perceptions of a patient’s age could vary.

*“Yes*,* when are you old? Eighty-five or older. Then there are patients who are very sick who look like they are eighty-five even though they are sixty-five*,* but what can you say—you are as frail as a sick*,* elderly person. I don’t think you’re old if you’re seventy-five to eighty. Definitely not! Not these days anyway. Maybe it was different in the past*,* but I don’t think that’s old.”* (Interview 4).

Conversely, situations such as cardiac arrest during elective surgery or in younger patients evoked a stronger inclination to persist, revealing how emotional and relational factors intersected with clinical reasoning. Familiarity with the patient or the presence of family members might also have prolonged efforts, highlighting how human connections and communication challenges subtly shaped decision-making. Terminating CPR in children and adolescents was perceived as difficult, even in cases where the prognosis and guidelines based on scientific evidence indicated that terminating CPR was medically appropriate.

*“I think*,* hand on heart*,* I think everyone struggles a little bit more the younger the person is. That you have a harder time maybe choosing to cancel CPR. Now I’m talking both hospital and pre-hospital. But there have been situations where you have had CPR on children or adolescents*,* and you can do CPR for hours. The chance of survival has long since disappeared*,* but no one wants to make the decision. I think that’s a big factor*,* that we have a harder time accepting the passing of a younger person.”* (Interview 11).

HCPs also noted that the presence or absence of family members could influence decision-making about when to terminate CPR. Treatment may have been prolonged if family members were present, and their absence could also have led to extended efforts when staff wanted to allow time for them to arrive or to provide information about the situation. Language barriers between HCPs and family members were described as another factor that could cause misunderstandings and hesitation in making decisions to end treatment.

Ultimately, this sub-theme illustrates that while guidelines aimed to ensure fairness, decision-making about continuing or terminating CPR were not purely technical. Instead, they were influenced by prognostic uncertainty, ethical considerations, and contextual factors that complicated the ideal of equality.

### Team decisions and communication – “Talking out loud to each other in the team”

Effective teamwork and open communication were critical for managing cardiac arrest. Regardless of whether care occurred in pre-hospital or in-hospital settings, the collective competence of the team was essential by combining medical knowledge, professional experience, and familiarity with cardiac arrest care. Effective communication fostered clarity, confidence, and a sense of security within the team. When roles were flexible and dialogue was open, collaboration was perceived as strong. However, the fact that team members often came from different units and may not knew each other well could complicate interactions, making explicit communication even more critical during patient handovers and treatment transitions.

Experience shaped team dynamics. Physicians with limited exposure to cardiac arrest may have hesitated or lost authority, prompting other team members such as specialist nurses with advanced life support skills, to assume greater responsibility. While this could ensure continuity of care, it also challenged traditional role boundaries and may have created tension. Leadership changes, such as when a physician arrived late, further underscored the need for clear communication and shared reasoning.

Decisions about terminating CPR were ideally made through dialogue, with the physician seeking consensus before acting. Openly stating the rationale for actions allowed the team to respond and align efforts. Yet, variations occured: some physicians made unilateral decisions, which could be difficult for others to accept and may have led to resistance. Confidence and experience appeared to influence decision-making styles; for example, experienced physicians were more likely to listen to the team and act decisively, which was valued by both nurses and physicians. If anyone objected, CPR often continued for a while longer until the topic was raised again.

“*It’s the physician who usually raises the question*,* but he mostly wants to confirm that what he thinks is right. We work a lot in teams*,* so it’s very good [to have dialogue]. If there’s anyone who says*,* ‘no I’m not comfortable with this*,*’ we’ll give it five more minutes. That’s how we discuss it. We’re at it a long time before we make that decision.*” (Interview 3).

Ultimately, this sub-theme illustrates that equality in care was not only about protocols but also about how teams communicated and collaborated under pressure. Transparent reasoning and mutual respect were key to ensuring that decision-making was ethically sound and collectively supported, especially when decisions concerned ending treatment.

### The importance of context – “The place contributes a lot”

The physical and social environment shaped how cardiac arrest care unfolded. Location influenced both practical and emotional aspects of treatment. In hospital settings, performing CPR in public spaces could be challenging, requiring efforts to shield the patient and maintain focus amid bystanders. These environmental factors added complexity to what was otherwise a highly standardized procedure.

*“Then it gets messy. You know you end up in the dining room. // We get an emergency alarm and then we go straight out. Then it depends. We don’t know what we will face or where it had happened.”* (Interview 3).

In out-of-hospital situations, context could affect the speed of diagnosis and initiation of CPR. Surroundings sometimes led to initial assumptions that delayed recognition; for example, in areas associated with substance use, non-responsiveness might first be attributed to intoxication rather than cardiac arrest. Nevertheless, once cardiac arrest was identified, CPR began promptly, underscoring the commitment to act despite contextual obstacles.

Ultimately, this sub-theme illustrates that while guidelines aimed to ensure equal care, the realities of space, environment, and social circumstances could influence how quickly and smoothly treatment unfolded.

### Reflection is needed – “Unfortunately you don’t really have the time”

This sub-theme underscores the tension between the urgency of cardiac arrest care and the need for professional reflection. Because CPR occured in highly time-critical situations, opportunities for reflection during the event were rare. In cases where cardiac arrest was anticipated, such as when the diagnosis was known before arrival, there may have been time for “reflection-in-action,” allowing HCPs to mentally prepare. More often, however, cardiac arrest was unexpected, and professionals acted immediately, setting emotions aside to focus on life-saving measures.

Post-event reflection was widely regarded as important for learning, emotional processing, and improving future decision-making, including discussions about CPR and DNAR orders. Yet, such reflection seldom occured, particularly when team members worked in different units or when organizational routines did not prioritize debriefing. HCPs described that early in their careers, reflection and debriefing were more common, but these practices tended to diminish over time. The absence of structured reflection could leave unresolved feelings, especially in cases where decisions to continue or terminate CPR were contested or regretted. In general, HCPs advocated that reflection should be carried out more often.

*“Actually*,* you should have a debriefing afterwards*,* but… The only time we had a debriefing*,* it was when I was a medical student*,* and it was because it was a young man who had had a cardiac arrest // but here I have never been through it. You would probably benefit from that.”* (Interview 12).

Occasionally, reflections touched on patient-related factors such as age, substance use, or socioeconomic status, though HCPs maintained that these did not influence CPR efforts. When socioeconomic aspects were mentioned, they were linked to lifestyle-related risk factors for cardiac arrest rather than to treatment decisions. Overall, this sub-theme revealed that while reflection was recognized as essential for ethical and professional growth, the pace and structure of emergency care often left little room for it.

## Discussion

HCPs in this study perceived that care for patients with sudden cardiac arrest is generally provided on equal terms, guided by strict protocols. They expressed that treatment is initiated for all patients regardless of gender, ethnicity, or socioeconomic status, and that decisions are rarely influenced by these factors. Whether these perceptions reflect actual practice cannot be determined from this study design. Previous research shows that ethnic disparities occur across conditions and settings [32], and cardiac arrest care is no exception: racial minorities have been found to receive inferior treatment and have lower survival rates in both out-of-hospital and in-hospital cardiac arrest [18], although findings are not uniform. Agerström et al. [19] reported higher survival among Middle Eastern patients compared to Nordic patients in Sweden [9]. Our findings suggest that ethnic discrimination is not readily acknowledged by HCPs, which aligns with studies indicating that such disparities often stem from unconscious bias rather than deliberate intent [33]. In pre-hospital contexts, ethnicity may indirectly affect care through cultural norms or family expectations that delay CPR initiation, particularly when gender roles or modesty norms complicate treatment.

Regarding gender, HCPs in the present study did not perceive any treatment disparities between men and women. This contrasts with evidence of gender disparities in other cardiac care domains, such as cardiac chest pain and cardiac troponin levels and the likelihood of receiving guideline-recommended care [34], identification of ST-elevation myocardial infarction [35], median door-to-STEMI activation [36], cardiac catheterization [37], admission to hospital for chest pain [38], and cardiac stress testing [39]. Studies on cardiac arrest care show divergent findings [40–42]. Overall, men appear more likely to survive out-of-hospital cardiac arrest [43, 44]. In contrast, recent research on in-hospital cardiac arrest found no treatment disparities but reported higher survival among women [12]. Possibly, this result can be partly explained by findings made by Perman et al. (2019) who confirmed the hypothesis that women have higher rates of DNAR orders. After adjusting for age, race, and comorbidity, women remained significantly more likely to have a DNAR order established during the first 24 h of their hospital admission after cardiac arrest [45]. In our study, participants rarely reported refraining from CPR, which may have influenced their perception of equality.

No discrimination based on SES was revealed in the present study, yet registry-based studies consistently show that lower SES is associated with poorer outcomes, including reduced likelihood of bystander CPR and survival [9, 22, 46–48]. Furthermore, previous research has found that higher education is associated with greater survival after out-of-hospital cardiac arrest [49]. These findings suggest that systemic factors, rather than individual intent, may drive SES-related disparities. Our results also indicate that perceived threats or unsafe environments in pre-hospital settings—often linked to socioeconomic conditions—can delay care, warranting further investigation.

In the present results, age was perceived as the most salient factor influencing decisions. Participants acknowledged that older patients may receive shorter CPR duration or be subject to DNAR orders without prior discussion, raising concerns about potential ageism. Although survival rates decline with age, evidence shows that older survivors can achieve good neurological outcome. Advanced age therefore does not preclude treatment with CPR [50, 51]. In a systematic review aiming to identify factors, facilitators, and barriers involved in DNAR decisions, older age was a contributory factor in 11 studies [52]. In a systematic review and meta-analysis of 10 studies, it was found that the use of DNAR orders increased with patient age. The review confirmed that age is an important determinant of the initiation of DNAR orders in critically ill patients, but whether this constitutes ageism remains unclear [53]. In a study of all patients who died in a Swedish community hospital, 630 patients had a documented DNAR order corresponding to 88.6% of the deceased. The DNAR orders had not been discussed with the patient/surrogate in almost three fourths of the patients, which raises questions about whether the decision to issue a DNAR order was partly related to the patient’s age [54]. If age alone determines CPR or DNAR decisions, this constitutes discrimination and challenges ethical principles of justice.

Finally, the participants were highly experienced and trained in advanced life support, which may explain their confidence in providing equal care. Previous research indicates that experience reduces stress and uncertainty during CPR. In a Swedish study of 2,154 HCPs, of whom 45% had previous real-life CPR experience, it was found that 61% of the HCPs would feel confident in their CPR knowledge, 86% would know what to do, and 60% would be able to take command if necessary. A short time since the latest real-life CPR performance and a high number of previous real-life CPR performances were associated with reduced likelihood of worrying about making mistakes, reduced likelihood of feeling stressed or anxious, and increased likelihood of feeling calm [55]. Less experienced HCPs might report different perceptions of discrimination, suggesting a need for comparative studies.

### Study limitations

A limitation in this study is the risk that those who were asked about discrimination against patients with cardiac arrest could have difficulty talking about the existence of discrimination. The willingness and ability to reflect on and talk about one’s own discrimination can be assumed to be limited and thereby weaken the trustworthiness of the study. However, requests to talk about discrimination that the participants have witnessed can be assumed to strengthen participants’ credibility.

The participants in this study had experience with varied contexts of cardiac arrest care and consisted of both nurses and physicians, which is a strength of the study. On the other hand, the transferability of the results can be assumed to be limited to healthcare contexts where HCPs are quite familiar with cardiac arrest events, given that the study participants are specialists in cardiac arrest care and used to having conversations about DNARs.

Thematic analysis was considered an appropriate method, partly for its flexibility [29] and its usefulness for summarizing key features of extensive data [56]. However, the method has also been criticized because flexibility can lead to inconsistency and a lack of coherence when developing themes [57].

Due to the COVID-19 pandemic, the interviews for this study were conducted digitally, which may be a limitation. When conducting interviews digitally it can be more difficult to create a natural conversation, and non-verbal expressions can be missed. On the other hand, it can be a flexible and safe way to conduct interviews that encourages interviewees to talk freely [58]. However, even though the participants had varied levels of experience managing digital platforms they all seemed to talk freely during the interviews and contributed rich material to the study.

The analysis was conducted in co-operation between the first and the last authors and then discussed in the full research group, which strengthens the credibility and dependability of the analysis [59]. In addition, the authors’ pre-understanding was discussed and reflected upon during the process, to decrease the risk of researcher bias.

## Conclusions

Healthcare professionals perceive cardiac arrest care as predominantly equal. However, the findings reveal subtle vulnerabilities that challenge this perception. Older age emerged as the most consistent factor influencing decisions, raising concerns about potential ageism and the need for clearer criteria and patient involvement in DNAR discussions.

Promoting equality in cardiac arrest care requires more than adherence to guidelines. It demands team-based competence, structured reflection after events, and proactive communication with patients about resuscitation preferences. These measures can help prevent unconscious bias and improve decision-making under pressure.

Future research should explore how age, multimorbidity, and psychiatric illness influence CPR decisions, examine the role of DNAR orders in practice, and investigate gender differences in survival and treatment. Understanding these dynamics is essential for developing interventions that safeguard equity in one of the most time-critical areas of healthcare.

## Supplementary Information

Supplementary Material 1.

Supplementary Material 2.

## Data Availability

The datasets generated and analysed during this study are not publicly available due to Swedish ethics legislation and the EU GDPR law but are available from the corresponding author upon reasonable request, if appropriate permissions are obtained from the Swedish authorities.

## Abbreviations

CPR: Cardiopulmonary Resuscitation
DNAR: Do not attempt resuscitationt
HCP: Health Care Professional
SES: Socioeconomic status
