# Supplementary material for: Equal cardiac arrest care — a qualitative study of healthcare professionals’ experiences
Source: BMC Med Ethics. 2026 Mar 5;27:76. doi: 10.1186/s12910-026-01428-0 (PMC13063511; doi:10.1186/s12910-026-01428-0)
Supplement: Supplementary file 1 — Supplementary Material 1. [file 12910_2026_1428_MOESM1_ESM.docx]

**Supplementary file 1**

**Interview guide-Equal cardiac arrest care (Individual interviews)**

The interview begins with a presentation of the study’s purpose and structure, followed by an open introductory question: Can you tell me about your experiences in caring for patients with sudden cardiac arrest?

Depending on what the person talks about, follow-up questions about equality will be asked to ensure that aspects of gender, age, ethnicity, and socioeconomic status are covered during the interview.

Follow-up questions of a reflective and circular nature will be asked:

- Can you tell me more…?
- You said that…, can you elaborate on that?
- What do you mean when you say…?
- Can you give an example?
- Can you describe more?
