# Supplementary material for: Equal cardiac arrest care — a qualitative study of healthcare professionals’ experiences
Source: BMC Med Ethics. 2026 Mar 5;27:76. doi: 10.1186/s12910-026-01428-0 (PMC13063511; doi:10.1186/s12910-026-01428-0)
Supplement: Supplementary file 2 — Supplementary Material 2. [file 12910_2026_1428_MOESM2_ESM.docx]

**COREQ (COnsolidated criteria for REporting Qualitative research) Checklist^[[1]](#endnote-1)^**

| **Topic** | **Item No.** | **Guide Questions/Description** | **Reported on page No.**  **OR**  **Not Applicable, (N/A)** |
| --- | --- | --- | --- |
| **Domain 1: Research team**  **and reflexivity** | | | |
| *Personal characteristics* | | | |
| Interviewer/facilitator | 1 | Which author/s conducted the interview or focus group? | AB and LÅ conducted the interviews  p.7 |
| Credentials | 2 | What were the researcher’s credentials? E.g. PhD, MD | All researchers are Phd  p.1 |
| Occupation | 3 | What was their occupation at the time of the study? | AB, JA and KÅ- professors at Linnaeus University  JI-RN and PhDat Kalmar County Hospital  LÅ- senior lecturer at Linnaeus University  p.1 |
| Gender | 4 | Was the researcher male or female? | JI, JA, KÅ, AB-male  LÅ-female  p. 7 |
| Experience and training | 5 | What experience or training did the researcher have? | All members of the research team were experienced researchers.  AB and LÅ, who conducted the interviews were both experienced researchers in qualitative methods  p.7 |
| *Relationship with*  *participants* | | | |
| Relationship established | 6 | Was a relationship established prior to study commencement? | Suitable HCPs were identified by a designated nurse and then contacted by email by the first and the last authors. The authors did not established any other relationship to the participants prior to study.  p.7 |
| Participant knowledge of  the interviewer | 7 | What did the participants know about the researcher? e.g. personal goals, reasons for doing the research | Before the interviews start the participants got written information about the study and also the names of the researchers. This information was repeated before the interview started and the researchers also gave some information about their role as researchers and their background as nurses.  p. 6 |
| Interviewer characteristics | 8 | What characteristics were reported about the interviewer/ facilitator? e.g. Bias, assumptions, reasons and interests in the research topic | p. 7 |
| **Domain 2: Study design** | | | |
| *Theoretical framework* | | | |
| Methodological orientation  and Theory | 9 | What methodological orientation was stated to underpin the study? e.g. grounded theory, discourse analysis, ethnography, phenomenology, content analysis | Reflexive thematic analysis  p. 5 & p.7 |
| *Participant selection* | | | |
| Sampling | 10 | How were participants selected? e.g. purposive, convenience, consecutive, snowball | Purposive sample  p. 6 |
| Method of approach | 11 | How were participants approached? e.g. face-to-face, telephone, mail, email | Face-to-face and via a digital platform  p.7 |
| Sample size | 12 | How many participants were in the study? | 12 participants  p. 6 |
| Non-participation | 13 | How many people refused to participate or dropped out? Reasons? | Two persons refused to participate due to a heavy workload.  p.6 |
| *Setting* | | | |
| Setting of data collection | 14 | Where was the data collected? e.g. home, clinic, workplace | At the participants workplace except one of the face-to-face interviews, which took place at the university.  p.7 |
| Presence of nonparticipants | 15 | Was anyone else present besides the participants and researchers? | No, no one else was present  p.7 |
| Description of sample | 16 | What are the important characteristics of the sample? e.g. demographic data, date | See table 1, p.8 |

| **Topic** | **Item No.** | **Guide Questions/Description** | **Reported**  **(Yes/No/Not Applicable, N/A)** |
| --- | --- | --- | --- |
| *Data collection* | | | |
| Interview guide | 17 | Were questions, prompts, guides provided by the authors? Was it pilot tested? | In supplementary file 1 the interview guide Is presented. |
| Repeat interviews | 18 | Were repeat inter views carried out? If yes, how many? | No repeated interviews were carried out.  p.6 |
| Audio/visual recording | 19 | Did the research use audio or visual recording to collect the data? | Audio recording  p. 7 |
| Field notes | 20 | Were field notes made during and/or after the interview or focus group? | p. 7 |
| Duration | 21 | What was the duration of the interviews or focus group? | The interview lasted between 34- 75 minutes. Described at p.7 |
| Data saturation | 22 | Was data saturation discussed? | p. 7 |
| Transcripts returned | 23 | Were transcripts returned to participants for comment and/or correction? | N/A |
| **Domain 3: Analysis and**  **findings** | | | |
| *Data analysis* | | | |
| Number of data coders | 24 | How many data coders coded the data? | Two researchers; LÅ & AB  p.7 |
| Description of the coding  tree | 25 | Did authors provide a description of the coding tree? | N/A |
| Derivation of themes | 26 | Were themes identified in advance or derived from the data? | Yes, themes derived from the data. This is described in phase 2 in the analysis process.  p. 7 |
| Software | 27 | What software, if applicable, was used to manage the data? | p. 7 |
| Participant checking | 28 | Did participants provide feedback on the findings? | N/A |
| *Reporting* | | | |
| Quotations presented | 29 | Were participant quotations presented to illustrate the themes/findings?  Was each quotation identified? e.g. participant number | Yes, presented in the results section, p. 8-15 |
| Data and findings consistent | 30 | Was there consistency between the data presented and the findings? | Yes |
| Clarity of major themes | 31 | Were major themes clearly presented in the findings? | Yes, p.8 |
| Clarity of minor themes | 32 | Is there a description of diverse cases or discussion of minor themes? | N/A |

1. Tong A, Sainsbury P, Craig J. Consolidated criteria for reporting qualitative research (COREQ): a 32-item checklist for interviews and focus groups. *International Journal for Quality in Health Care*. 2007. Volume 19, Number 6: pp. 349 – 357. [↑](#endnote-ref-1)
